# Evaluating the Visibility of Power-Line Bird-Collision Warning Devices Using an Avian Spectral-Response Weighting Model

**DOI:** 10.3390/ani16162562

**Published:** 2026-08-17

**Authors:** Mengxuan Li, Wenbin Li, Geng Huang, Yanjun Kuang, Zhi Yang, Jing Hu, Yifei Jia

**Affiliations:** 1 State Grid Electric Power Engineering Research Institute Co., Ltd., Beijing 100069, Chinayangzhi0713@foxmail.com (Z.Y.); 2 School of Ecology and Nature Conservation, Beijing Forestry University, Beijing 100083, China; 3 Centre for East Asian-Australasian Flyway Studies, Beijing Forestry University, Beijing 100083, China; 4 State Grid Jiangxi Electric Power Research Institute, Nanchang 330096, China; kuangyj0101@126.com (Y.K.);

**Keywords:** bird collision, power line, warning device, multispectral imaging, visibility, collision mitigation

## Abstract

Collisions with overhead power lines are an important source of avian mortality, particularly for migratory and large-bodied birds. Although warning devices are widely installed to increase line visibility, their image-based visibility across viewing distances and directions remains poorly quantified. We evaluated eight power-line bird-collision warning devices using UAV-based multispectral imaging and an avian spectral-response weighting model. Images were acquired at 5, 10, 20, 30, and 50 m under upward-looking, horizontal, and downward-looking viewing geometries. A composite visibility score quantified target–background separation within the multispectral images. Raw mean visibility increased from 0.351 at 5 m to 0.484 at 50 m; however, the distance effect was not significant after accounting for viewing angle and heterogeneous residual variance. Viewing angle was the dominant source of variation at 20–50 m, with substantially higher visibility under downward-looking views than under horizontal and upward-looking views. A significant distance × viewing-angle interaction further showed that distance-response patterns differed among viewing geometries: visibility increased with distance under downward-looking views but remained consistently low under horizontal and upward-looking views. The relative performance of the tested devices also changed under low-visibility conditions, with the self-luminous tag and circular tag B attaining the highest condition-standardized mean scores. These findings identify viewing geometry as a critical determinant of warning-device visibility and demonstrate that averages pooled across observation conditions can obscure important performance differences. Evaluation and deployment should therefore prioritize device visibility from below and during near-horizontal viewing, particularly for overhead ground or shield wires at medium and long distances. This condition-specific framework provides a quantitative basis for improving the design and placement of power-line bird-collision warning devices.

## Simple Summary

Birds can collide with overhead power lines when the wires or installed warning devices are difficult to see against the surrounding background. We examined how viewing distance and direction affected the visibility of eight commonly used warning-device designs. Images were collected with a drone-mounted camera that recorded several bands of visible and near-infrared light at distances from 5 to 50 m. Device visibility was measured from the difference between each device and its background in the images. Viewing direction had a stronger influence than distance alone. Devices were generally much more visible when viewed from above, whereas visibility remained low when they were viewed from below or at nearly the same height. The relative performance of the tested devices also changed under these more difficult viewing conditions, with the self-luminous tag and circular tag B producing the highest visibility measures. These findings show that warning devices should be evaluated under the viewing directions and backgrounds most relevant to bird collision risk. Considering visibility from below and during near-horizontal approaches may improve the design and placement of warning devices, support bird conservation, and help power-line managers select devices more effectively.

## 1. Introduction

Bird collisions with overhead power lines are a widely recognized conservation issue worldwide. As electricity transmission infrastructure continues to expand, power lines increasingly cross wetlands, river valleys, grasslands, forest edges, agricultural landscapes, and migratory corridors, where they overlap spatially with bird migration routes, foraging areas, commuting pathways, and local movement spaces. Power lines can affect birds and other wildlife through collision, electrocution, habitat alteration, barrier effects, and corridor effects, with collision mortality representing one of the most direct and extensively documented impacts [1,2,3,4]. The risk of bird collisions with power lines is not determined solely by line distribution or bird activity intensity; it is also shaped by species traits, flight behavior, habitat type, topographic context, light conditions, and the height and diameter of conductors and ground wires [5,6,7,8]. Therefore, evaluating mitigation measures in relation to bird behavior and visual perception is therefore important for improving the ecological design and management of electricity infrastructure [9,10,11].

Installing warning devices on conductors or ground wires is currently one of the most widely used measures to mitigate bird collisions with power lines. Devices such as wire markers, hanging plates, flight diverters, reflective or light-emitting devices, and aviation warning balls are designed to enhance the visual detectability of linear structures against environmental backgrounds, allowing birds to identify potential obstacles before reaching the line and to adjust their flight paths accordingly [12,13,14,15]. Field studies and meta-analyses have shown that wire-marking measures can generally reduce bird collisions, although their effectiveness varies with species composition, line configuration, marker type, deployment design, landscape context, and monitoring methods [16,17,18,19].

Birds and humans differ in photoreceptor composition, spectral sensitivity, oil-droplet filtering, and sensitivity to ultraviolet wavelengths. These differences can affect how brightness, color, and contrast relationships between warning devices and environmental backgrounds are represented [20,21,22,23]. Receptor-noise-limited and spectral-sensitivity models further show that visual discrimination depends on both the spectral properties of the target and background and the response characteristics of the observer’s visual system [24,25,26]. From a sensory–ecology perspective, the visibility of a human-made structure also depends on its position within the visual field and the viewing geometry of an approaching bird [27,28]. These findings have encouraged the development of bird-oriented approaches to warning-device design, including visual signals that differ from those most apparent to humans [29,30].

The visual signal produced by a warning device is also expected to vary with observation geometry. As a bird approaches a power line, viewing distance, projected target size, and background composition change continuously. Close-range visibility describes device–background separation near the line, whereas visibility at medium and long distances indicates whether a visual signal is potentially available earlier in the approach. Viewing direction may be equally important because upward, horizontal, and downward lines of sight place the same device against different combinations of sky, vegetation, terrain, and infrastructure. Limited sensitivity to achromatic contrast may further reduce the potential detectability of warning devices against low-contrast backgrounds [31]. Distance and viewing direction should therefore be evaluated jointly when comparing the visual performance of warning devices.

Despite growing evidence that line marking can reduce collision mortality, standardized image-based comparisons of warning devices remain limited. Existing assessments often rely on human visual judgment, close-range observations, or field collision outcomes that integrate multiple ecological and engineering processes. Consequently, it remains unclear how image-derived visibility changes jointly with viewing distance and direction, and whether device rankings are maintained under low-visibility conditions. Here, visibility refers to the image-derived separation of a warning device from its background, whereas detectability refers to the potential availability of that visual signal to an approaching bird. Avoidance behavior and collision reduction are considered distinct outcomes requiring field evaluation.

We combined UAV-based multispectral imaging with an avian spectral-response weighting model to evaluate eight warning-device specimens across five viewing distances and eight observation geometries. Specifically, we examined (1) whether image-derived visibility varied with distance after accounting for viewing-angle differences; (2) how visibility differed among viewing-angle groups at medium and long distances, and whether device rankings changed under upward and horizontal low-visibility conditions; and (3) whether distance-response patterns differed among viewing-angle groups. This framework provides a standardized basis for comparing warning devices across observation conditions relevant to their screening, deployment, and further optimization.

## 2. Methods

### 2.1. Warning Devices, Observation Design, and Multispectral Image Acquisition

**Figure 1:**
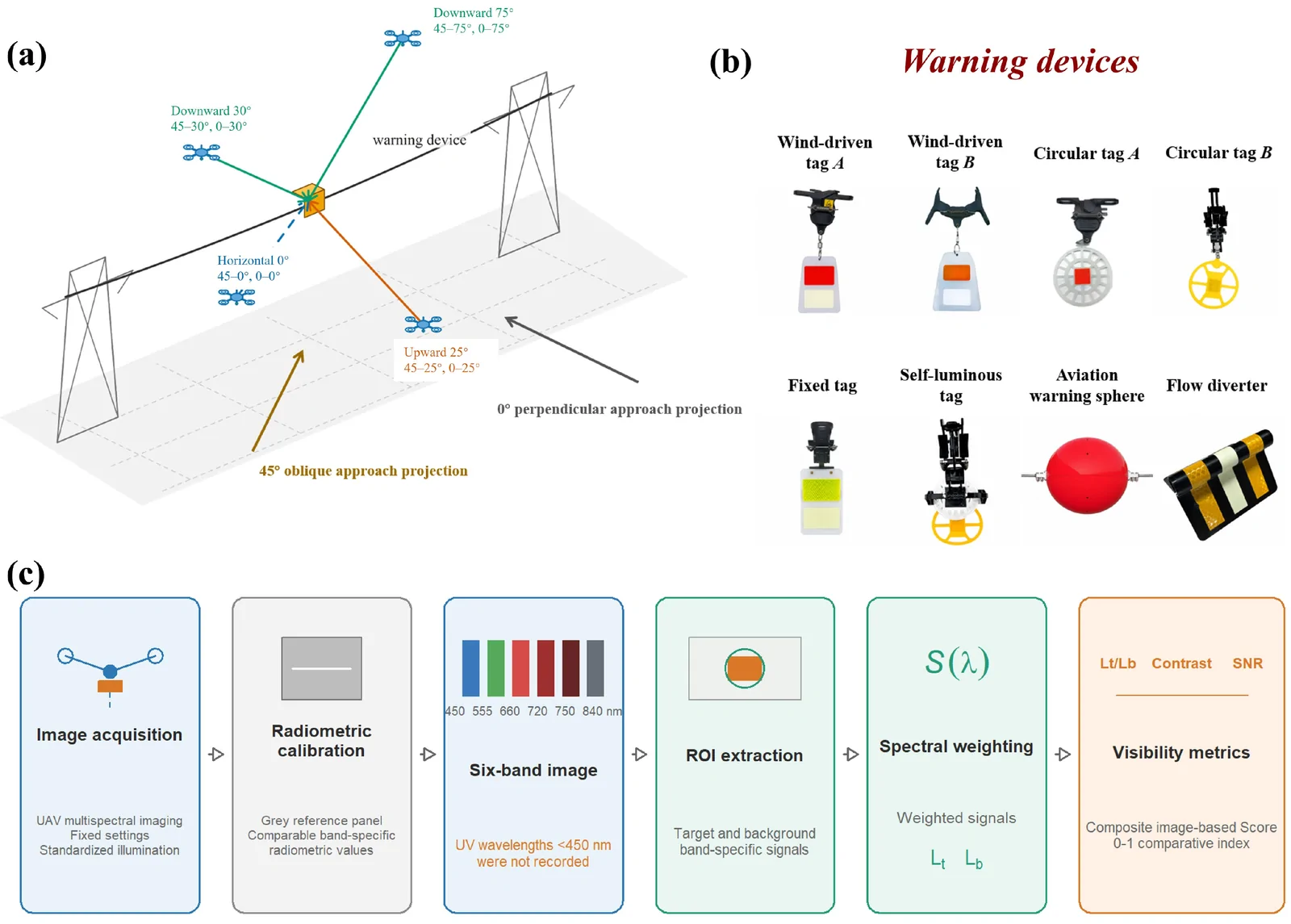
Experimental design, warning devices, and multispectral image-processing workflow used to evaluate the visibility of power-line bird-collision warning devices. (**a**) Schematic representation of the upward-looking, horizontal, and downward-looking viewing geometries used during image acquisition. The 0° and 45° lines indicate the perpendicular and oblique ground-projection directions, respectively. (**b**) The eight warning-device designs evaluated in this study. (**c**) Conceptual workflow showing standardized UAV-based multispectral acquisition; grey-panel radiometric calibration; extraction of target and background regions of interest; generalized spectral-response weighting of the six recorded bands; calculation of weighted target and background signals; and derivation of the component metrics and composite image-based visibility score.

Eight warning-device designs commonly used on overhead power lines were evaluated: a flow diverter, a fixed tag, two wind-driven tags (A and B), two circular tags (A and B), a self-luminous tag, and an aviation warning sphere (Figure 1b). One physical specimen of each design was used for image acquisition, and each specimen was photographed separately under the prescribed observation conditions.

Multispectral images were acquired in Chengdu, China, using a DJI Matrice 350 RTK unmanned aerial vehicle (DJI, Shenzhen, China) equipped with a YUSENSE MS600 Pro multispectral camera (Yusense, Qingdao, China) (Figure S1). The principal specifications of the imaging platform and acquisition settings are summarized in Table 1, with full manufacturer specifications provided in Table S1. Image acquisition was conducted February 2026 between 09:00 and 11:00 and between 13:00 and 16:00, under sunny to lightly cloudy conditions, with sufficient natural illumination and no perceptible wind (Figure S5). A manufacturer-supplied grey reference panel (Yusense, Qingdao, China) was used for radiometric calibration following the camera-operating protocol. Imaging settings were held constant throughout the experiment, except for the prescribed changes in viewing orientation. Five consecutive images were acquired for each device–distance–viewing-direction combination, while the device position and surrounding scene remained unchanged.

Images were collected at camera-to-device distances of 5, 10, 20, 30, and 50 m. The 5–10 m observations represented close-range conditions, whereas 20–50 m represented medium-to-long-range conditions relevant to advance visual detection. This distance series allowed device visibility to be evaluated across progressive changes in projected target size and target–background composition.

Viewing geometry was defined by the horizontal approach projection and the vertical orientation of the camera-to-device line of sight (Figure 1a). Horizontal projections of 0° and 45° represented perpendicular and oblique observations relative to the power line, respectively. Four vertical orientations were examined within each horizontal projection: horizontal (0°), upward-looking (25°), moderately downward-looking (30°), and steeply downward-looking (75°). Their combination produced eight observation geometries: 0–0°, 0–25°, 0–30°, 0–75°, 45–0°, 45–25°, 45–30°, and 45–75°. For statistical analysis, the two 25° geometries were classified as upward, the two 0° geometries as horizontal, and the four 30° and 75° geometries as downward.

### 2.2. Avian Spectral-Response Weighting and Visibility Metrics

Radiometrically calibrated multispectral images were processed using an avian spectral-response weighting workflow (Figure 1c). For each image, the warning device and its adjacent background were delineated as target and background regions, respectively, and radiance values were extracted from the six bands centered at 450, 555, 660, 720, 750, and 840 nm. The spectral radiance of each region was weighted using a generalized avian spectral-response function, $S\left(\lambda \right)$. The weighted target and background luminance signals were calculated as follows:

$$L_{t}^{*}=\int_{450}^{840}L_{t}\left(\lambda \right)S\left(\lambda \right)d\lambda$$

$$L_{b}^{*}=\int_{450}^{840}L_{b}\left(\lambda \right)S\left(\lambda \right)d\lambda$$

where $L_{t}\left(\lambda \right)$ and $L_{b}\left(\lambda \right)$ are the target and background radiance at wavelength λ, respectively. The integrals were numerically approximated using the six measured spectral bands. The target-to-background luminance ratio was subsequently calculated as $L_{t}^{*}/L_{b}^{*}$.

The generalized response function provided a common wavelength-dependent weighting for standardized comparisons among warning devices and observation conditions. The continuous weighted signals were approximated from the six measured spectral bands. Because the sensor did not record wavelengths below 450 nm, ultraviolet signals were not represented, and the model could not distinguish between ultraviolet-sensitive and violet-sensitive avian visual systems. The resulting signals should therefore be interpreted as generalized avian-response-weighted engineering measures of relative target–background separation within the recorded spectral range, rather than as species-specific estimates of perceived brightness or color contrast.

Six image-derived metrics were calculated from the spectrally weighted target and background values. The target-to-background luminance ratio ($L_{t}^{*}/L_{b}^{*}$) described the relative luminance of the device against its background. Target luminance variability ($std_{t}$) and background clutter intensity ($std_{bg}$) were calculated as the sample standard deviations of pixel-level weighted luminance within the respective regions:

$$std_{t}=\sqrt{\frac{1}{N-1}\sum_{i=1}^{N}{\left(L_{ti}-\bar{L_{t}}\right)}^{2}}$$

$$std_{bg}=\sqrt{\frac{1}{M-1}\sum_{j=1}^{M}{\left(L_{bj}-\bar{L_{b}}\right)}^{2}}$$

where *N* and *M* are the numbers of target and background pixels, $L_{ti}$ and $L_{bj}$ are their weighted luminance values, and ($\bar{L_{t}}$) and ($\bar{L_{b}}$) are the corresponding mean values.

Normalized target–background contrast was calculated as follows:

$$Contrast=\frac{\left|\bar{L_{t}}-\bar{L_{b}}\right|}{\bar{L_{t}}+\bar{L_{b}}}$$

Signal-to-noise ratio was calculated as follows:

$$SNR=\frac{\left|\bar{L_{t}}-\bar{L_{b}}\right|}{\sqrt{\sigma_{t}^{2}+\sigma_{b}^{2}}}$$

where $\sigma_{t}$ and $\sigma_{b}$ are the standard deviations of weighted luminance within the target and background regions. Absolute differences were used so that both brighter and darker targets could generate positive contrast and signal-separation values.

The overall visibility score (Score) combined the luminance ratio, normalized contrast, and SNR:

$$Score=0.3\left(\frac{L_{t}/L_{b}}{L_{t}/L_{b}+1}\right)+0.4\left(Contrast\right)+0.3\left(\frac{SNR}{SNR+1}\right)$$

The transformations $x/\left(x+1\right)$ mapped the unbounded luminance-ratio and SNR components onto the interval [0, 1). Component weights were specified a priori using the predefined engineering evaluation framework described in the Supplementary Methods and were not estimated from the present dataset [31,32]. The overall Score was bounded between 0 and 1, with higher values indicating stronger image-based separation between a tested device and its selected background under the corresponding acquisition condition. Score was used as the primary comparative response variable, whereas the remaining metrics described individual contributions from relative luminance, target variability, background structure, normalized contrast, and signal-to-noise separation. The component weights and composite Score were not calibrated against avian detection thresholds, avoidance responses, or collision outcomes.

The generalized model rests on four assumptions. First, a single spectral-response function provides consistent comparative weighting but does not represent any particular bird species. Second, the six sensor bands adequately approximate target and background signals within 450–840 nm, while finer spectral features and wavelengths below 450 nm remain unresolved. Third, grey-panel calibration and standardized acquisition support stable relative comparisons among images. Fourth, the selected target and background regions and fixed metric weights provide a consistent index of device–background separation. These assumptions limit transferability across species, habitats, and field conditions. The model is therefore intended for standardized screening, not for predicting species-specific perception, avoidance behavior, or collision-risk reduction.

### 2.3. Data Processing and Statistical Analysis

The overall visibility score (Score) was used as the primary response variable (Table S3). For each combination of device, distance, and viewing direction, five images were acquired consecutively under unchanged conditions. Score was calculated for each image and averaged across the five technical replicates; only the condition-level mean was retained. The statistical unit was therefore the condition mean rather than the individual image. After quality control, 265 records were available, including 263 observations within 5–50 m and 152 observations at medium and long distances (20–50 m) (Table S2).

Each of the eight device designs was represented by one physical specimen that was repeatedly imaged across experimental conditions. Distance and viewing-angle effects were analyzed using linear mixed-effects models, with tested device identity included as a random intercept to account for repeated observations of the same specimen. Viewing distance was treated as a categorical fixed factor because no linear response to distance was assumed, and viewing-angle group was included when estimating the overall distance effect. Model assumptions were assessed using residual, quantile–quantile, and variance diagnostic plots. Because residual variance differed among viewing-angle groups, the primary models estimated separate residual variances for upward, horizontal, and downward observations. An equal-variance model and a model treating device identity as a fixed blocking factor were used as sensitivity analyses (Figures S2–S4).

The viewing-angle effect was evaluated at 20–50 m using a heteroscedastic mixed-effects model with viewing-angle group and distance as fixed factors. Model-adjusted means and 95% confidence intervals were estimated for each viewing-angle group. Pairwise contrasts were adjusted using the Holm procedure, and partial η^2^ was reported as an effect-size measure.

The distance × viewing-angle interaction was evaluated by comparing an additive model containing the main effects of distance and viewing angle with a model estimating a separate mean for each observed combination of distance and viewing angle. Both models included a random intercept for tested device and viewing-angle-specific residual variances. The models were fitted by maximum likelihood and compared using a likelihood-ratio test. This approach avoided estimating the unobserved upward-viewing condition at 50 m. The change in marginal R^2^ was used to quantify the additional variation explained by the interaction. Robustness was evaluated using two subsets with complete or near-complete coverage: all three viewing-angle groups at 5–20 m, and horizontal and downward observations at 5–50 m. Additional sensitivity analyses used log-transformed distance, logit-transformed Score, and exclusion of observations with absolute normalized residuals greater than three.

Because each device design was represented by only one specimen, device design and specimen identity were completely confounded. Device performance was therefore compared descriptively rather than through population-level tests of device type. Analyses were conducted separately for all viewing angles and for low-visibility conditions, defined as upward and horizontal observations at 20–50 m. Score was first averaged within each combination of device, distance, and viewing angle. Only combinations represented for all eight specimens were retained and assigned equal weight when calculating condition-standardized device means. Ranking stability was evaluated by sequentially omitting each shared condition.

Finally, response-scale sensitivity was assessed by repeating the principal analyses after logit, square-root, square, arcsine-square-root, and rank-normalizing transformations of Score. These analyses evaluated whether the viewing-angle effect, distance × viewing-angle interaction, and low-visibility device rankings were robust to the numerical scale of the response variable. Mixed-effects models were fitted in R 4.5.1 using the nlme and emmeans packages. Statistical significance was assessed at *p* < 0.05.

## 3. Results

### 3.1. Overall Visibility Increased Descriptively with Viewing Distance

**Figure 2:**
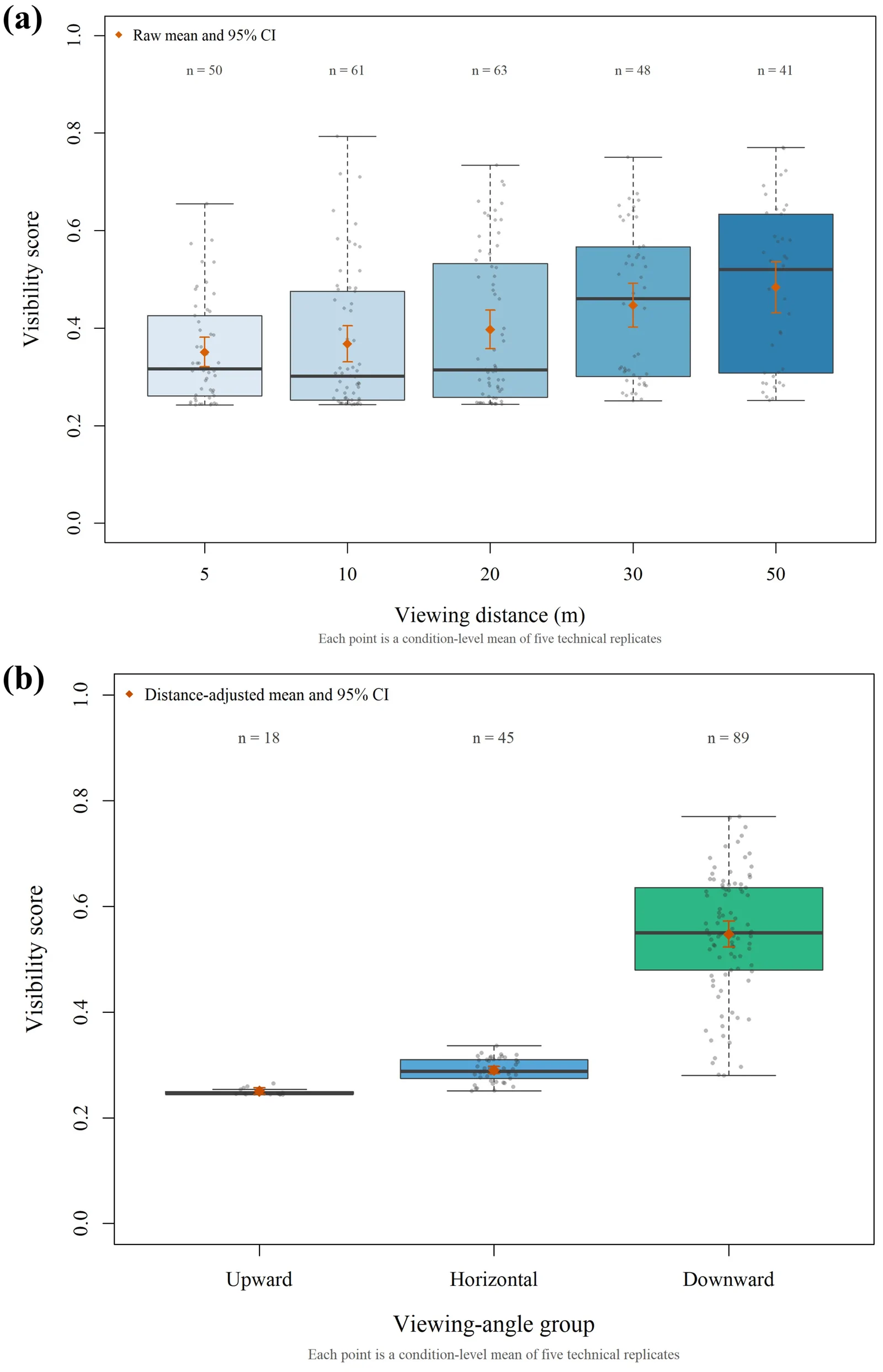
Variation in warning-device visibility across viewing distances and viewing-angle groups. (**a**) Distribution of visibility scores at viewing distances of 5–50 m. Orange diamonds and error bars indicate raw means and 95% confidence intervals, respectively. (**b**) Distribution of visibility scores among upward, horizontal, and downward viewing-angle groups at 20–50 m. Orange diamonds and error bars indicate distance-adjusted means and 95% confidence intervals estimated using the heteroscedastic linear mixed-effects model. Boxes show the interquartile range, center lines indicate medians, whiskers extend to 1.5 times the interquartile range, and grey points represent condition-level means of five consecutive technical replicates. Values above the boxes indicate sample sizes.

Across the 5–50 m observation range, the raw mean visibility score increased from 0.351 at 5 m to 0.368 at 10 m, 0.397 at 20 m, 0.447 at 30 m, and 0.484 at 50 m (Figure 2a). The corresponding 95% confidence intervals were 0.321–0.381, 0.331–0.405, 0.358–0.437, 0.402–0.492, and 0.431–0.536, respectively. Score distributions nevertheless overlapped substantially among distances, particularly between adjacent distance classes.

Variation among observations also increased with distance. The standard deviation rose from 0.107 at 5 m to 0.166 at 50 m, and the interquartile range broadened at medium and long distances. Thus, the increase in the raw mean was accompanied by greater variation among combinations of device and viewing condition.

After heterogeneity in residual variance among viewing-angle groups was accounted for, the mixed-effects model did not support a common effect of distance across viewing directions (F (4, 249) = 1.91, *p* = 0.109, and partial η^2^ = 0.030). By comparison, an equal-variance model indicated a significant distance effect (F (4, 249) = 4.73, *p* = 0.001, partial η^2^ = 0.071). The absence of a common distance effect in the heteroscedastic model was consistent across alternative transformations of Score. Consequently, the raw increase in visibility with distance should not be interpreted as a uniform response across viewing directions; direction-specific distance responses are evaluated through the distance × viewing-angle interaction in Section 3.4.

### 3.2. Viewing Angle Dominated Visibility Differences at Medium and Long Distances

Viewing-angle group had a pronounced effect on visibility at 20–50 m (Figure 2b). After adjustment for viewing distance and repeated observations of the same tested device, the estimated mean Score was 0.251 for upward views (95% CI: 0.245–0.256; *n* = 18), 0.290 for horizontal views (95% CI: 0.284–0.297; *n* = 45), and 0.548 for downward views (95% CI: 0.523–0.572; *n* = 89).

The heteroscedastic mixed-effects model identified a strong viewing-angle effect (F (2, 140) = 286.77, *p* < 0.001, and partial η^2^ = 0.804). Holm-adjusted comparisons showed that visibility was higher for downward than for upward or horizontal views, and higher for horizontal than for upward views (all adjusted *p* < 0.001). The adjusted mean differences were 0.297 between downward and upward views, 0.257 between downward and horizontal views, and 0.040 between horizontal and upward views.

Viewing direction was therefore the strongest measured source of variation in visibility at medium and long distances. Scores remained consistently low for upward and horizontal views, whereas downward views produced substantially higher and more variable scores.

### 3.3. Device Rankings Shifted Under Low-Visibility Viewing Conditions

**Figure 3:**
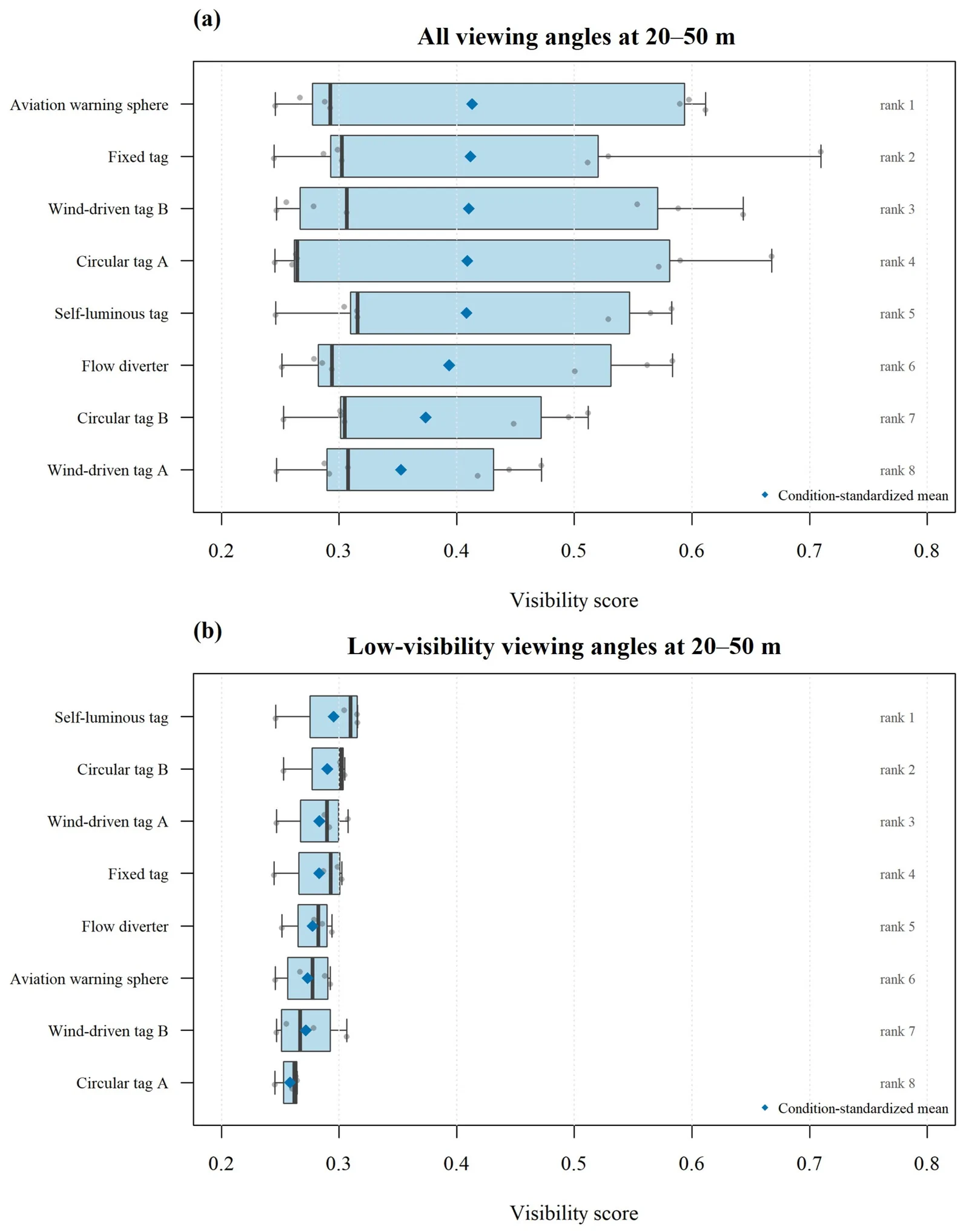
Condition-standardized visibility of the eight tested warning-device specimens under all-view and low-visibility conditions at 20–50 m. (**a**) Visibility across all viewing-angle groups, standardized over seven distance-by-viewing-angle conditions shared by all tested devices. (**b**) Visibility under low-visibility viewing angles (upward and horizontal), standardized over four shared conditions. Boxes show the interquartile range, center lines indicate medians, whiskers extend to 1.5 times the interquartile range, and grey points represent condition-specific means. Blue diamonds indicate equally weighted condition-standardized means. Rankings describe the relative performance of the tested specimens rather than population-level differences among device types.

The relative rankings of the eight tested device specimens differed between the all-view and low-visibility analyses (Figure 3). Across seven combinations of distance and viewing angle shared by all devices, the aviation warning sphere had the highest condition-standardized Score (0.413), followed by the fixed tag (0.412), wind-driven tag B (0.410), circular tag A (0.409), and self-luminous tag (0.408). The flow diverter (0.394), circular tag B (0.374), and wind-driven tag A (0.353) ranked lower. The broad overlap among condition-level distributions and the leave-one-condition-out analysis showed that the leading all-view rankings were sensitive to individual viewing conditions (Figure 3a).

A different and more stable ranking emerged across the four shared low-visibility conditions represented by upward and horizontal views (Figure 3b). The self-luminous tag had the highest condition-standardized Score (0.295), followed by circular tag B (0.290), wind-driven tag A (0.283), and the fixed tag (0.283), whereas circular tag A had the lowest value (0.258). The self-luminous tag, circular tag B, and circular tag A retained ranks 1, 2, and 8, respectively, whenever one shared condition was omitted.

Performance averaged across all viewing directions therefore did not reproduce the rankings observed under low-visibility conditions. Because each design was represented by one physical specimen, these rankings describe the tested devices rather than population-level differences among device types.

### 3.4. The Distance × Viewing-Angle Interaction Revealed Different Distance-Response Patterns Among Approach Directions

**Figure 4:**
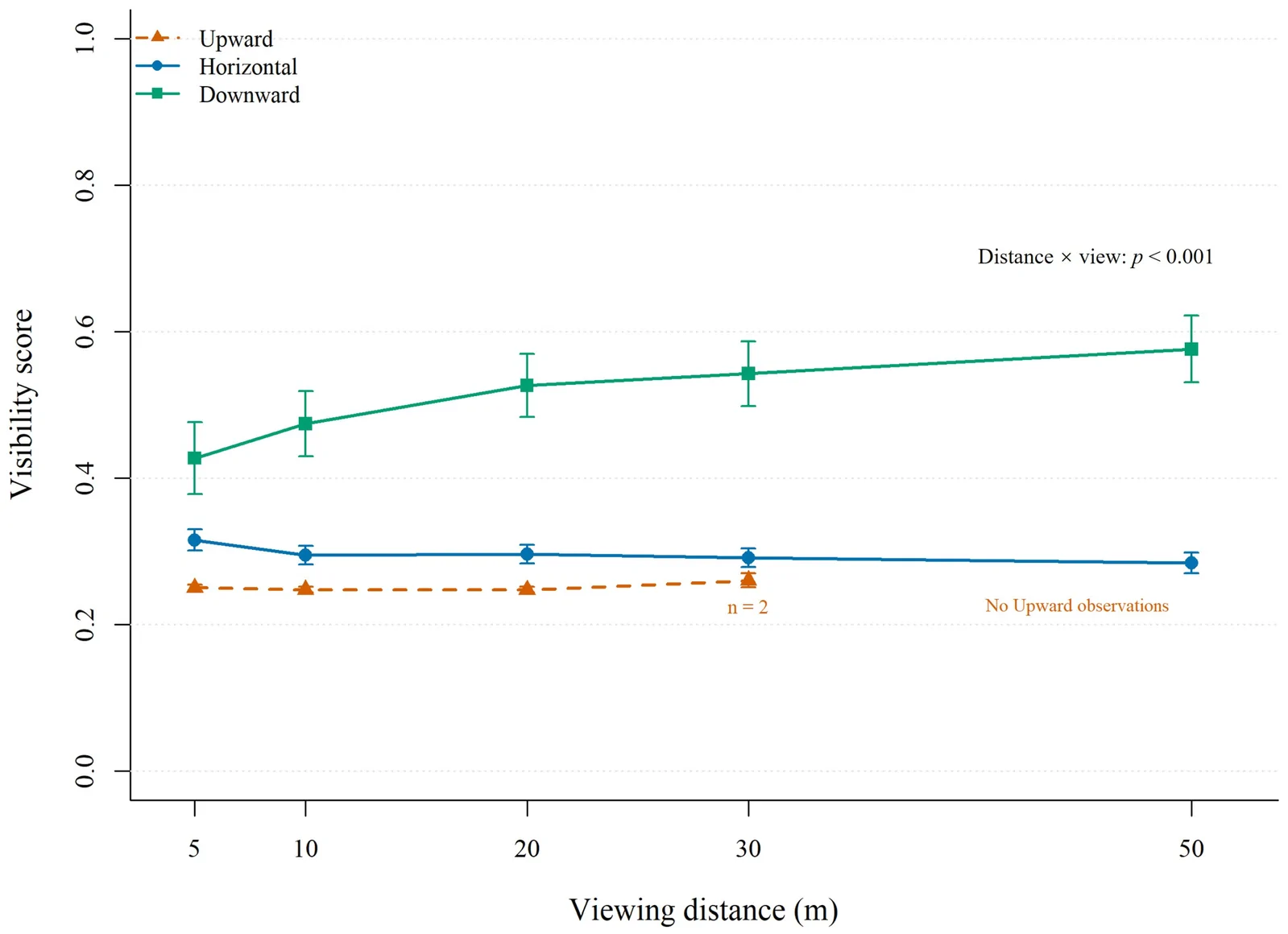
Distance-dependent changes in warning-device visibility across viewing-angle groups. Points represent model-estimated mean visibility scores, and error bars indicate 95% confidence intervals.

The effect of viewing distance differed among viewing-angle groups (Figure 4). A mixed model estimating separate means for the observed combinations of distance and viewing angle fitted the data better than an additive model containing only their main effects (likelihood-ratio test: χ^2^ (7) = 35.65, *p* < 0.001). Including the interaction increased the marginal R^2^ by 0.055.

Downward visibility increased with distance, from an estimated mean Score of 0.427 at 5 m to 0.474 at 10 m, 0.527 at 20 m, 0.542 at 30 m, and 0.576 at 50 m. In contrast, horizontal visibility remained low and declined slightly from 0.315 at 5 m to 0.284 at 50 m. Upward visibility was consistently lowest and changed little between 5 and 20 m (0.250–0.247). The estimate at 30 m was 0.260 but was based on only two observations, and no upward observations were available at 50 m.

The interaction was also supported in the complete 5–20 m subset containing all three viewing-angle groups (*p* = 0.017) and in the 5–50 m subset restricted to horizontal and downward views (*p* < 0.001). Models using log-transformed distance, employing logit-transformed Score, or excluding observations with large normalized residuals produced consistent results. The overall increase in raw visibility with distance was therefore primarily associated with downward viewing rather than a common increase across approach directions.

## 4. Discussion

### 4.1. Approach Distance and Viewing Angle Jointly Determine the Early Visibility of Warning Devices

The raw mean visibility Score increased from 5 to 50 m, but the heteroscedastic mixed-effects model did not identify a common distance effect across viewing directions. Instead, the significant distance × viewing-angle interaction showed that the apparent distance pattern arose from contrasting responses among viewing-angle groups. Visibility increased progressively with distance under downward views, remained low and changed little under horizontal views, and was consistently lowest under upward views. Thus, the overall increase in the raw mean reflected the composition of viewing conditions rather than a uniform improvement in device visibility with distance.

This distinction is important when evaluating warning devices. Viewing distance changes projected target size, but it can also alter the proportion and spectral composition of the background surrounding the device. These effects may operate differently when a device is viewed against the ground, vegetation, sky, or other line structures. Distance should therefore be interpreted together with viewing direction and target-background configuration rather than as an isolated determinant of visibility.

The 20–50 m range remains relevant for comparing the potential availability of visual signals before birds reach the immediate vicinity of a power line. Visual cues available at these distances may provide more opportunity for flight-path adjustment than cues that emerge only at close range [27,32]. Previous studies have similarly emphasized that warning-device evaluation should consider whether visual information becomes available at distances compatible with obstacle detection and flight control [33]. Our findings add that the availability of an image-based signal at a given distance depends strongly on viewing direction. However, the present experiment did not measure whether birds detected the devices or changed their trajectories. Medium- and long-distance performance should therefore be interpreted as potential signal availability within defined viewing conditions, not as demonstrated avoidance distance.

### 4.2. Low-Visibility Viewing Angles Reveal Device-Type Differences

Viewing angle was the strongest measured source of variation at 20–50 m. Downward views produced substantially higher visibility than horizontal and upward views, while upward views consistently yielded the lowest Scores. These differences likely reflect changes in both observation geometry and environmental background. A device viewed from above may be projected against terrain or vegetation with relatively strong target–background separation, whereas the same device viewed from below or near horizontally may produce a weaker visual signal.

The relative rankings of the eight tested specimens also changed between the all-view and low-visibility analyses. The aviation warning sphere and fixed tag ranked highly when all viewing conditions were combined, whereas the self-luminous tag and circular tag B ranked highest under upward and horizontal views. Circular tag A showed the lowest standardized Score under these low-visibility conditions. These shifts indicate that an all-view average may not represent device performance under the viewing directions where image-derived visibility is most constrained. Because each design was represented by one physical specimen, these rankings characterize the tested devices and provide hypotheses for broader comparisons involving multiple specimens of each design.

A plausible collision pathway may occur where birds encounter vertically separated line structures. An approaching bird may first detect the larger phase conductors and initiate an upward flight adjustment. During this maneuver, the thinner overhead ground or shield wire may remain difficult to distinguish from below or from a near-horizontal line of sight. The bird could then enter the space occupied by the upper wire despite responding to the lower conductors. The persistently low upward and horizontal Scores are consistent with the visual geometry expected in this scenario. However, confirming this pathway requires direct measurements of bird trajectories, detection events, and avoidance maneuvers.

The tested devices that retained relatively higher visibility under low-visibility views may have produced more stable luminance differences, stronger target–background contrast, or more persistent outlines across observation conditions. Visual target detection depends on whether luminance and color differences provide a signal above the relevant perceptual threshold [22], and recent evidence indicates that many birds have limited sensitivity to targets with low achromatic contrast [34]. Device development could therefore focus on maintaining target–background contrast, outline stability, reflective differences, or active light signals under upward and near-horizontal views. These design features should be evaluated across representative backgrounds rather than inferred from general appearance to human observers.

### 4.3. Implications for Evaluation, Deployment, and Field Validation

The results support condition-specific screening of bird-collision warning devices for power lines. In wetlands, river corridors, farmland, migratory bottlenecks, and routes between roosting and foraging sites, screening should consider flight directions, line configuration, dominant backgrounds, and ground-wire position. Spatial risk mapping and bird-activity data can first identify priority line sections. Image-based evaluation can then compare candidate devices under the viewing conditions expected at those locations [35]. Particular attention should be given to upper ground or shield wires viewed from below. A higher Score indicates stronger image-based target–background separation under the tested conditions, but it does not demonstrate earlier detection or reduced collision risk. Field studies show that line marking can reduce bird collisions; however, effectiveness varies among species, devices, and environmental settings [36]. The method is therefore best used to select candidates for subsequent behavioral and field testing. The examined distances describe camera-to-device observation conditions and do not determine marker spacing. Installation intervals should remain based on field evidence, focal species, line characteristics, and applicable technical guidance [37,38,39].

Interpretation of the generalized spectral-response model depends on four principal assumptions. First, the six calibrated bands between 450 and 840 nm are assumed to sample the target and background differences relevant to the comparative analysis. This assumption excludes ultraviolet information below 450 nm and may be less transferable to ultraviolet-sensitive taxa or ultraviolet-reflective materials. Second, the generalized response function is assumed to provide a common weighting suitable for standardized comparisons among devices. It does not reproduce species-specific cone sensitivities, ocular-media transmission, oil-droplet filtering, receptor noise, or neural processing. Third, static target–background separation is assumed to represent the availability of a potential visual signal. This approximation does not include movement, temporal integration, visual-field position, attention, or behavior. Fourth, the a priori component weights in Score are assumed to represent the relative contributions of luminance ratio, contrast, and SNR. Because these weights were not calibrated against avian responses, Score should be interpreted as a comparative engineering index rather than a perceptual or behavioral threshold. Together, these assumptions support comparisons under standardized conditions but limit direct transfer to species-specific field effectiveness.

The distinction between ultraviolet-sensitive (UVS) and violet-sensitive (VS) systems illustrates why species-specific transfer cannot be assumed. Many raptors have VS-type SWS1 systems, whereas UVS systems are widespread among passerines and parrots [40]. Variation also occurs within collision-relevant shorebirds. Gulls possess UVS-type opsins, whereas wood sandpipers, common sandpipers, red phalaropes, and common snipes have been classified as VS [41]. UVS and VS birds differ in the peak sensitivity of the SWS1 cone and in the transmission of short wavelengths through the ocular media. Consequently, the same device-background spectrum may generate different receptor signals among taxa. Species also differ in cone distributions, visual acuity, visual-field structure, flight speed, and maneuverability. The patterns identified here should therefore guide taxon-specific comparisons rather than serve as a universal ranking of warning devices.

Environmental conditions further limit transfer from standardized images to operational power lines. Radiometric calibration provided a common basis for comparison, but field signals may change with weather, ambient illumination, sky brightness, vegetation, water reflections, seasonal backgrounds, device movement, material ageing, and nighttime conditions. Testing across these sources of variation is necessary to determine whether the low visibility observed under upward and horizontal views persists across habitats and deployment periods.

Three priorities follow from these limitations. First, species-specific visual models should incorporate measured reflectance, ultraviolet wavelengths, focal-species cone sensitivities, ocular-media transmission, and receptor-noise parameters. Second, controlled behavioral experiments should test whether model-derived Scores predict detection distance, attention, or avoidance responses. Third, field validation should combine bird-trajectory monitoring with before–after collision surveys to determine whether improved image-based separation corresponds to fewer collisions. These steps would connect standardized device screening to biological effectiveness while preserving the distinction between modeled visual contrast and demonstrated conservation outcomes.

## 5. Conclusions

This study demonstrates that viewing geometry is a primary determinant of the image-based visibility of power-line bird-collision warning devices. Distance responses differed markedly among viewing directions: visibility increased progressively with distance under downward views, whereas upward and horizontal views remained consistently low across the tested range. This distance × viewing-angle interaction shows that warning-device visibility cannot be adequately characterized by distance or pooled averages alone. Instead, upward and near-horizontal viewing conditions define a persistent visibility bottleneck at medium and long distances.

The relative rankings of the tested devices also changed under these low-visibility conditions. The self-luminous tag and circular tag B maintained the highest standardized Scores under upward and horizontal views, demonstrating the value of evaluating devices under the conditions in which visual separation from the background is most constrained. These results provide a practical basis for prioritizing upward and near-horizontal performance when screening warning devices, particularly for overhead ground or shield wires viewed from below. More broadly, the UAV-based multispectral framework developed here establishes a standardized approach for identifying direction-specific visibility differences and supports the development and deployment of warning devices tailored to high-risk power-line configurations.

## Figures and Tables

**Table 1:** Technical specifications of the UAV–multispectral imaging system and image-acquisition settings.

Component	Parameter	Specification
UAV platform	Model	DJI Matrice 350 RTK
	Positioning system	Real-time kinematic (RTK) positioning
Imaging payload	Model	YUSENSE MS600 Pro multispectral camera
	Sensor size	1/3 inch
	Spectral channels	6
	Effective pixels	1.2 megapixels
	Radiometric resolution	12 bit
	Field of view	49.5° × 38.1°
	Ground sampling distance	8.65 cm at 120 m above ground level
	Swath width	110 m × 83 m at 120 m above ground level
Spectral configuration	Central wavelength/bandwidth	450/30, 555/27, 660/22, 720/10, 750/10 and 840/30 nm
	Spectral coverage	Visible and near-infrared wavelengths; no ultraviolet band recorded
Calibration	Radiometric reference	Manufacturer-supplied grey reference panel
Experimental objects	Warning devices	8 designs, represented by one tested specimen per design
Observation design	Camera-to-target distance	5, 10, 20, 30, and 50 m
	Horizontal approach projection	0° perpendicular and 45° oblique to the power line
	Vertical viewing orientation	0° horizontal, 25° upward-looking, and 30° and 75° downward-looking
Image acquisition	Technical replicates	5 consecutive images per experimental condition
	Camera settings	Held constant except for the prescribed viewing orientation
Field conditions	Location	Chengdu, China
	Time of day	09:00–11:00 and 13:00–16:00
	Weather	Sunny to lightly cloudy, adequate illumination, and no detectable wind

## Data Availability

The original contributions presented in this study are included in the article/Supplementary Materials. Further inquiries can be directed to the corresponding authors.

## Supplementary Materials

The following supporting information can be downloaded at: https://www.mdpi.com/article/10.3390/ani16162562/s1. Figure S1: UAV-based multispectral imaging platform used for image acquisition. The DJI Matrice 350 RTK carried a YUSENSE MS600 Pro multispectral camera; Table S1: Full manufacturer specifications of the UAV platform and multispectral payload; Table S2: Number of condition-level mean Score records by viewing distance and viewing-angle group within the 5–50 m analytical range; Table S3: Condition-level visibility Score dataset used in this study; Figure S2: Diagnostic plots for the heteroscedastic linear mixed-effects model used to evaluate the overall distance effect. Panels show normalized residuals against fitted values, a normal Q–Q plot, the residual distribution, and normalized residuals by viewing distance; Figure S3: Diagnostic plots for the heteroscedastic linear mixed-effects model used to evaluate the viewing-angle effect at 20–50 m. Panels show normalized residuals against fitted values, a normal Q–Q plot, the residual distribution, and normalized residuals by viewing-angle group; Figure S4: Diagnostic plots for the mixed-effects model used to evaluate the distance × viewing-angle interaction. Panels show normalized residuals against fitted values, a normal Q–Q plot, the residual distribution, and normalized residuals by viewing-angle group; Figure S5: Representative pseudocolored images of warning devices following multispectral processing. The displayed colors visualize differences in the processed spectral signals and do not represent subjective avian color appearance.
