# Supplementary material for: Evaluating the Visibility of Power-Line Bird-Collision Warning Devices Using an Avian Spectral-Response Weighting Model
Source: Animals (Basel). 2026 Aug 17;16(16):2562. doi: 10.3390/ani16162562 (PMC13508915; doi:10.3390/ani16162562)
Supplement: Supplementary file 1 [file animals-16-02562-s001.zip › animals-4430953-supplementary.pdf]

Supplementary Material

Evaluating the Visibility of Power-Line Bird-Collision Warning Devices Using an Avian Spectral-Response Weighting Model

Supplementary Methods

S1. Field setup and imaging platform

Multispectral images were acquired using a DJI Matrice 350 RTK unmanned aerial vehicle carrying a YUSENSE MS600 Pro multispectral payload (Figure S1). The principal acquisition settings are reported in the main text, while the complete manufacturer specifications of the platform and payload are provided in Table S1.

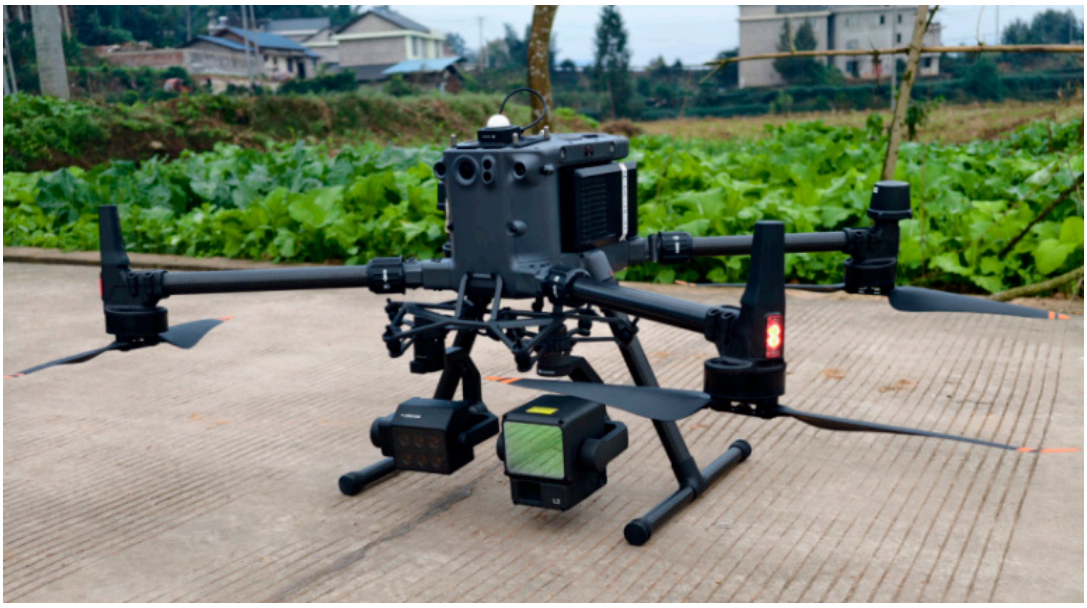

Figure S1. UAV-based multispectral imaging platform used for image acquisition. The DJI Matrice 350 RTK carried a YUSENSE MS600 Pro multispectral camera.

Table S1. Full manufacturer specifications of the UAV platform and multispectral payload.

Component	Parameter	Specification
UAV platform	Model	DJI Matrice 350 RTK
UAV platform	Dimensions, unfolded	810 × 670 × 430 mm (L × W × H), excluding propellers
UAV platform	Dimensions, folded	430 × 420 × 430 mm (L × W × H), including propellers
UAV platform	Diagonal wheelbase	895 mm
UAV platform	Weight	Approximately 3.77 kg without batteries; approximately 6.47 kg with two TB65 batteries
UAV platform	Maximum take-off weight	9.2 kg
UAV platform	Hovering accuracy	Vertical: ±0.1 m (vision), ±0.5 m (GNSS), ±0.1 m (RTK); horizontal: ±0.3 m (vision), ±1.5 m (GNSS), ±0.1 m (RTK)
UAV platform	RTK positioning accuracy	1 cm + 1 ppm horizontally; 1.5 cm + 1 ppm vertically (RTK FIX)
UAV platform	Maximum angular velocity	Pitch: 300° s^{-1}; yaw: 100° s^{-1}
UAV platform	Maximum aircraft pitch angle	30°; 25° in N mode with the forward vision system enabled
UAV platform	Maximum ascent speed	6 m s^{-1}
UAV platform	Maximum vertical descent speed	5 m s^{-1}
UAV platform	Maximum tilted descent speed	7 m s^{-1}
UAV platform	Maximum horizontal speed	23 m s^{-1}
UAV platform	Maximum wind-speed resistance	12 m s^{-1}
UAV platform	Maximum flight time	55 min under manufacturer test conditions
Multispectral payload	Model	YUSENSE MS600 Pro
Multispectral payload	Configuration	6 multispectral channels
Multispectral payload	Sensor size	1/3 inch
Multispectral payload	Effective pixels	1.2 megapixels
Multispectral payload	Shutter	Global shutter
Multispectral payload	Radiometric resolution	12 bit
Multispectral payload	Field of view	49.5° × 38.1°
Multispectral payload	Ground sampling distance	8.65 cm at 120 m above ground level
Multispectral payload	Swath width	110 m × 83 m at 120 m above ground level
Multispectral payload	Spectral bands	450 nm/30 nm, 555 nm/27 nm, 660 nm/22 nm, 720 nm/10 nm, 750 nm/10 nm and 840 nm/30 nm (central wavelength/bandwidth)
Multispectral payload	Optical window	Sapphire optical-glass window
Multispectral payload	Dimensions	≤130 × 160 × 150 mm with the optical axis oriented vertically downward
Multispectral payload	Weight	≤670 g

Component	Parameter	Specification
Multispectral payload	Mounting and power interface	DJI X-Port
Multispectral payload	Image format	16-bit raw TIFF and 8-bit reflectance JPEG, including GPS and ambient-light information
Multispectral payload	Storage	64 GB microSD supplied; supports up to 128 GB, U3 rating or higher
Multispectral payload	Processing software	Yusense Map / Yusense Map Plus
Multispectral payload	Parameter control	DJI Pilot
Multispectral payload	Trigger modes	Overlap-based, timed and flight-controller triggering
Multispectral payload	Maximum capture frequency	1 Hz
Multispectral payload	Operating temperature	−10 to 50 °C, with relative airflow ≥1 m s^{-1}
Multispectral payload	Storage temperature	−30 to 70 °C
Multispectral payload	Operating humidity	≤85% relative humidity, non-condensing
Multispectral payload	Certifications	CE, FCC and RoHS

Note: UAV specifications are based on the official DJI Matrice 350 RTK technical specifications. Payload specifications are based on the YUSENSE MS600 Pro product specification sheet. Values describe manufacturer-rated performance and not necessarily the conditions achieved during this experiment.

S2. Condition-level dataset and sample coverage

Five images were acquired consecutively for each tested device–distance–viewing-direction combination without repositioning the device or changing the surrounding scene. Score was calculated for each image and averaged across the five technical replicates. The resulting condition-level mean was used as the statistical unit. The complete dataset contained 265 condition means. Two exploratory observations acquired at 100 m were retained in Table S3 for data transparency but were excluded from the predefined 5–50 m analyses, leaving 263 records for the principal analyses.

The distribution of the 263 analytical records across distance and viewing-angle groups is summarized in Table S2. Sampling was complete or nearly complete for horizontal and downward views, whereas upward observations were limited to two records at 30 m and were unavailable at 50 m.

Table S2. Number of condition-level mean Score records by viewing distance and viewing-angle group within the 5–50 m analytical range.

Viewing-angle group	5 m	10 m	20 m	30 m	50 m	Total
Upward	14	16	16	2	0	48
Horizontal	12	16	16	16	13	73
Downward	24	29	31	30	28	142
Total	50	61	63	48	41	263

Note: Each record is the mean of five consecutive technical replicates.

Table S3. Condition-level visibility Score dataset used in this study.

Tested device	Viewing direction	Distance (m)	Viewing-angle group	Score
Flow diverter	0–0°	5	Horizontal	0.328200
Flow diverter	0–25°	5	Upward	0.272967
Flow diverter	0–30°	5	Downward	0.437367
Flow diverter	0–75°	5	Downward	0.479400
Flow diverter	45–0°	5	Horizontal	0.292200
Flow diverter	45–25°	5	Upward	0.256400
Flow diverter	45–30°	5	Downward	0.386767
Flow diverter	45–75°	5	Downward	0.361167
Flow diverter	0–0°	10	Horizontal	0.290067
Flow diverter	0–25°	10	Upward	0.266633
Flow diverter	0–30°	10	Downward	0.457933
Flow diverter	0–75°	10	Downward	0.517400
Flow diverter	45–0°	10	Horizontal	0.287500
Flow diverter	45–25°	10	Upward	0.250200
Flow diverter	45–30°	10	Downward	0.374100

Tested device	Viewing direction	Distance (m)	Viewing-angle group	Score
Flow diverter	45–75°	10	Downward	0.397600
Flow diverter	0–0°	20	Horizontal	0.293633
Flow diverter	0–25°	20	Upward	0.256500
Flow diverter	0–30°	20	Downward	0.477833
Flow diverter	0–75°	20	Downward	0.595300
Flow diverter	45–0°	20	Horizontal	0.294100
Flow diverter	45–25°	20	Upward	0.246300
Flow diverter	45–30°	20	Downward	0.460067
Flow diverter	45–75°	20	Downward	0.469267
Flow diverter	0–0°	30	Horizontal	0.287200
Flow diverter	0–30°	30	Downward	0.529500
Flow diverter	0–75°	30	Downward	0.675800
Flow diverter	45–0°	30	Horizontal	0.284400
Flow diverter	45–30°	30	Downward	0.532300
Flow diverter	45–75°	30	Downward	0.510067
Flow diverter	0–0°	50	Horizontal	0.278700
Flow diverter	0–30°	50	Downward	0.537200
Flow diverter	0–75°	50	Downward	0.714300
Flow diverter	45–30°	50	Downward	0.555300
Flow diverter	45–75°	50	Downward	0.527600
Flow diverter	45–30°	100	Downward	0.441600
Fixed tag	0–0°	5	Horizontal	0.385500
Fixed tag	0–25°	5	Upward	0.250850
Fixed tag	0–30°	5	Downward	0.573300
Fixed tag	0–75°	5	Downward	0.361700
Fixed tag	45–0°	5	Horizontal	0.302300
Fixed tag	45–25°	5	Upward	0.242600
Fixed tag	45–30°	5	Downward	0.535700
Fixed tag	45–75°	5	Downward	0.260850
Fixed tag	0–0°	10	Horizontal	0.307400
Fixed tag	0–25°	10	Upward	0.246100
Fixed tag	0–30°	10	Downward	0.582950
Fixed tag	0–75°	10	Downward	0.479050
Fixed tag	45–0°	10	Horizontal	0.325950
Fixed tag	45–25°	10	Upward	0.242900
Fixed tag	45–30°	10	Downward	0.571500
Fixed tag	45–75°	10	Downward	0.251950
Fixed tag	0–0°	20	Horizontal	0.293650
Fixed tag	0–25°	20	Upward	0.244900
Fixed tag	0–30°	20	Downward	0.622450
Fixed tag	0–75°	20	Downward	0.504800
Fixed tag	45–0°	20	Horizontal	0.311150
Fixed tag	45–25°	20	Upward	0.244150
Fixed tag	45–30°	20	Downward	0.621550
Fixed tag	45–75°	20	Downward	0.296650

Tested device	Viewing direction	Distance (m)	Viewing-angle group	Score
Fixed tag	0–0°	30	Horizontal	0.292450
Fixed tag	0–30°	30	Downward	0.648200
Fixed tag	0–75°	30	Downward	0.525800
Fixed tag	45–0°	30	Horizontal	0.281300
Fixed tag	45–30°	30	Downward	0.628650
Fixed tag	45–75°	30	Downward	0.313250
Fixed tag	0–0°	50	Horizontal	0.309700
Fixed tag	0–30°	50	Downward	0.722600
Fixed tag	0–75°	50	Downward	0.635400
Fixed tag	45–0°	50	Horizontal	0.288000
Fixed tag	45–30°	50	Downward	0.770600
Wind-driven tag A	0–0°	5	Horizontal	0.296700
Wind-driven tag A	0–25°	5	Upward	0.243900
Wind-driven tag A	0–30°	5	Downward	0.535400
Wind-driven tag A	0–75°	5	Downward	0.328900
Wind-driven tag A	45–0°	5	Horizontal	0.312000
Wind-driven tag A	45–25°	5	Upward	0.248300
Wind-driven tag A	45–30°	5	Downward	0.434000
Wind-driven tag A	45–75°	5	Downward	0.262500
Wind-driven tag A	0–0°	10	Horizontal	0.272500
Wind-driven tag A	0–25°	10	Upward	0.244200
Wind-driven tag A	0–30°	10	Downward	0.482400
Wind-driven tag A	0–75°	10	Downward	0.301500
Wind-driven tag A	45–0°	10	Horizontal	0.278000
Wind-driven tag A	45–25°	10	Upward	0.246200
Wind-driven tag A	45–30°	10	Downward	0.307500
Wind-driven tag A	45–75°	10	Downward	0.266800
Wind-driven tag A	0–0°	20	Horizontal	0.274200
Wind-driven tag A	0–25°	20	Upward	0.244400
Wind-driven tag A	0–30°	20	Downward	0.558100
Wind-driven tag A	0–75°	20	Downward	0.354700
Wind-driven tag A	45–0°	20	Horizontal	0.309400
Wind-driven tag A	45–25°	20	Upward	0.249300
Wind-driven tag A	45–30°	20	Downward	0.386200
Wind-driven tag A	45–75°	20	Downward	0.373400
Wind-driven tag A	0–0°	30	Horizontal	0.297400
Wind-driven tag A	0–30°	30	Downward	0.504100
Wind-driven tag A	0–75°	30	Downward	0.482600
Wind-driven tag A	45–0°	30	Horizontal	0.317600
Wind-driven tag A	45–30°	30	Downward	0.450100
Wind-driven tag A	45–75°	30	Downward	0.341900
Wind-driven tag A	0–0°	50	Horizontal	0.287400
Wind-driven tag A	0–30°	50	Downward	0.480000
Wind-driven tag A	0–75°	50	Downward	0.587800
Wind-driven tag A	45–30°	50	Downward	0.391800

Tested device	Viewing direction	Distance (m)	Viewing-angle group	Score
Wind-driven tag A	45–75°	50	Downward	0.429100
Circular tag A	0–0°	5	Horizontal	0.272400
Circular tag A	0–25°	5	Upward	0.244100
Circular tag A	0–30°	5	Downward	0.580300
Circular tag A	0–75°	5	Downward	0.395900
Circular tag A	45–0°	5	Horizontal	0.308500
Circular tag A	45–25°	5	Upward	0.242500
Circular tag A	45–30°	5	Downward	0.655000
Circular tag A	45–75°	5	Downward	0.313000
Circular tag A	0–0°	10	Horizontal	0.252600
Circular tag A	0–25°	10	Upward	0.243600
Circular tag A	0–30°	10	Downward	0.577100
Circular tag A	0–75°	10	Downward	0.566500
Circular tag A	45–0°	10	Horizontal	0.255500
Circular tag A	45–25°	10	Upward	0.243800
Circular tag A	45–30°	10	Downward	0.709900
Circular tag A	45–75°	10	Downward	0.257200
Circular tag A	0–0°	20	Horizontal	0.264500
Circular tag A	0–25°	20	Upward	0.244800
Circular tag A	0–30°	20	Downward	0.631000
Circular tag A	0–75°	20	Downward	0.641700
Circular tag A	45–0°	20	Horizontal	0.256100
Circular tag A	45–25°	20	Upward	0.245800
Circular tag A	45–30°	20	Downward	0.734200
Circular tag A	45–75°	20	Downward	0.280400
Circular tag A	0–0°	30	Horizontal	0.262000
Circular tag A	0–30°	30	Downward	0.640400
Circular tag A	0–75°	30	Downward	0.665700
Circular tag A	45–0°	30	Horizontal	0.266600
Circular tag A	45–30°	30	Downward	0.750200
Circular tag A	45–75°	30	Downward	0.303800
Circular tag A	0–0°	50	Horizontal	0.268000
Circular tag A	0–30°	50	Downward	0.652000
Circular tag A	0–75°	50	Downward	0.582800
Circular tag A	45–0°	50	Horizontal	0.259000
Circular tag A	45–30°	50	Downward	0.768300
Self-luminous tag	0–0°	5	Horizontal	0.328100
Self-luminous tag	0–25°	5	Upward	0.243900
Self-luminous tag	0–30°	5	Downward	0.493850
Self-luminous tag	0–75°	5	Downward	0.485500
Self-luminous tag	45–0°	5	Horizontal	0.328250
Self-luminous tag	45–25°	5	Upward	0.244250
Self-luminous tag	45–30°	5	Downward	0.470300
Self-luminous tag	45–75°	5	Downward	0.425500
Self-luminous tag	0–0°	10	Horizontal	0.312200

**Tested device**	**Viewing direction**	**Distance (m)**	**Viewing-angle group**	**Score**
Self-luminous tag	0–25°	10	Upward	0.244450
Self-luminous tag	0–30°	10	Downward	0.486600
Self-luminous tag	0–75°	10	Downward	0.517800
Self-luminous tag	45–0°	10	Horizontal	0.319650
Self-luminous tag	45–25°	10	Upward	0.245150
Self-luminous tag	45–30°	10	Downward	0.449600
Self-luminous tag	45–75°	10	Downward	0.435750
Self-luminous tag	0–0°	20	Horizontal	0.312150
Self-luminous tag	0–25°	20	Upward	0.245550
Self-luminous tag	0–30°	20	Downward	0.552500
Self-luminous tag	0–75°	20	Downward	0.568750
Self-luminous tag	45–0°	20	Horizontal	0.319200
Self-luminous tag	45–25°	20	Upward	0.246850
Self-luminous tag	45–30°	20	Downward	0.489250
Self-luminous tag	45–75°	20	Downward	0.505850
Self-luminous tag	0–0°	30	Horizontal	0.316850
Self-luminous tag	0–30°	30	Downward	0.543750
Self-luminous tag	0–75°	30	Downward	0.621100
Self-luminous tag	45–0°	30	Horizontal	0.313750
Self-luminous tag	45–30°	30	Downward	0.550050
Self-luminous tag	45–75°	30	Downward	0.544650
Self-luminous tag	0–0°	50	Horizontal	0.301100
Self-luminous tag	0–30°	50	Downward	0.518750
Self-luminous tag	0–75°	50	Downward	0.691800
Self-luminous tag	45–0°	50	Horizontal	0.307800
Self-luminous tag	45–30°	50	Downward	0.577250
Self-luminous tag	45–75°	50	Downward	0.543250
Circular tag B	0–0°	5	Horizontal	0.320050
Circular tag B	0–25°	5	Upward	0.245950
Circular tag B	0–30°	5	Downward	0.347350
Circular tag B	0–75°	5	Downward	0.412600
Circular tag B	45–0°	5	Horizontal	0.312600
Circular tag B	45–25°	5	Upward	0.267850
Circular tag B	45–30°	5	Downward	0.444950
Circular tag B	45–75°	5	Downward	0.275300
Circular tag B	0–0°	10	Horizontal	0.317650
Circular tag B	0–25°	10	Upward	0.245400
Circular tag B	0–30°	10	Downward	0.315650
Circular tag B	0–75°	10	Downward	0.440400
Circular tag B	45–0°	10	Horizontal	0.284250
Circular tag B	45–25°	10	Upward	0.256150
Circular tag B	45–30°	10	Downward	0.475450
Circular tag B	45–75°	10	Downward	0.286150
Circular tag B	0–0°	20	Horizontal	0.322700
Circular tag B	0–25°	20	Upward	0.246150

Tested device	Viewing direction	Distance (m)	Viewing-angle group	Score
Circular tag B	0–30°	20	Downward	0.399350
Circular tag B	0–75°	20	Downward	0.587650
Circular tag B	45–0°	20	Horizontal	0.287050
Circular tag B	45–25°	20	Upward	0.259500
Circular tag B	45–30°	20	Downward	0.525800
Circular tag B	45–75°	20	Downward	0.281800
Circular tag B	0–0°	30	Horizontal	0.320350
Circular tag B	0–30°	30	Downward	0.440350
Circular tag B	0–75°	30	Downward	0.627800
Circular tag B	45–0°	30	Horizontal	0.282800
Circular tag B	45–30°	30	Downward	0.565750
Circular tag B	45–75°	30	Downward	0.346500
Circular tag B	0–0°	50	Horizontal	0.315950
Circular tag B	0–30°	50	Downward	0.459650
Circular tag B	0–75°	50	Downward	0.642100
Circular tag B	45–0°	50	Horizontal	0.285850
Circular tag B	45–30°	50	Downward	0.579800
Circular tag B	45–75°	50	Downward	0.365200
Wind-driven tag B	0–25°	5	Upward	0.255800
Wind-driven tag B	45–25°	5	Upward	0.244100
Wind-driven tag B	0–0°	10	Horizontal	0.278900
Wind-driven tag B	0–25°	10	Upward	0.245700
Wind-driven tag B	0–30°	10	Downward	0.640500
Wind-driven tag B	45–0°	10	Horizontal	0.377200
Wind-driven tag B	45–25°	10	Upward	0.244200
Wind-driven tag B	45–30°	10	Downward	0.793500
Wind-driven tag B	45–75°	10	Downward	0.482100
Wind-driven tag B	0–0°	20	Horizontal	0.277000
Wind-driven tag B	0–25°	20	Upward	0.247500
Wind-driven tag B	0–30°	20	Downward	0.693300
Wind-driven tag B	0–75°	20	Downward	0.655700
Wind-driven tag B	45–0°	20	Horizontal	0.336400
Wind-driven tag B	45–25°	20	Upward	0.246000
Wind-driven tag B	45–30°	20	Downward	0.700800
Wind-driven tag B	45–75°	20	Downward	0.523900
Wind-driven tag B	0–0°	30	Horizontal	0.251000
Wind-driven tag B	0–30°	30	Downward	0.568100
Wind-driven tag B	0–75°	30	Downward	0.651500
Wind-driven tag B	45–0°	30	Horizontal	0.305700
Wind-driven tag B	45–30°	30	Downward	0.662300
Wind-driven tag B	45–75°	30	Downward	0.471100
Wind-driven tag B	0–30°	50	Downward	0.547700
Wind-driven tag B	0–75°	50	Downward	0.633500
Wind-driven tag B	45–0°	50	Horizontal	0.255400
Wind-driven tag B	45–30°	50	Downward	0.643300

Tested device	Viewing direction	Distance (m)	Viewing-angle group	Score
Wind-driven tag B	45–75°	50	Downward	0.389300
Aviation warning sphere	0–0°	10	Horizontal	0.300200
Aviation warning sphere	0–25°	10	Upward	0.252400
Aviation warning sphere	45–0°	10	Horizontal	0.255800
Aviation warning sphere	45–25°	10	Upward	0.244600
Aviation warning sphere	45–30°	10	Downward	0.716200
Aviation warning sphere	45–75°	10	Downward	0.613500
Aviation warning sphere	0–0°	20	Horizontal	0.270400
Aviation warning sphere	0–25°	20	Upward	0.244300
Aviation warning sphere	0–30°	20	Downward	0.539200
Aviation warning sphere	45–0°	20	Horizontal	0.314400
Aviation warning sphere	45–25°	20	Upward	0.247200
Aviation warning sphere	45–30°	20	Downward	0.635900
Aviation warning sphere	45–75°	20	Downward	0.660100
Aviation warning sphere	0–0°	30	Horizontal	0.266100
Aviation warning sphere	0–25°	30	Upward	0.253900
Aviation warning sphere	0–30°	30	Downward	0.547400
Aviation warning sphere	45–0°	30	Horizontal	0.310000
Aviation warning sphere	45–25°	30	Upward	0.265500
Aviation warning sphere	45–30°	30	Downward	0.632200
Aviation warning sphere	0–0°	50	Horizontal	0.251400
Aviation warning sphere	0–30°	50	Downward	0.520600
Aviation warning sphere	45–0°	50	Horizontal	0.282400
Aviation warning sphere	45–30°	50	Downward	0.674400
Aviation warning sphere	0–30°	100	Downward	0.487200

Note: Viewing direction is expressed as horizontal approach projection–vertical line-of-sight orientation. Each Score is the mean of five consecutive technical replicates. The two observations at 100 m were exploratory and were not included in the 5–50 m principal analyses.

S3. Mixed-effects model diagnostics

Model adequacy was evaluated using normalized residuals. For each primary mixed-effects model, residuals were examined against fitted values, with normal Q–Q plots and histograms used to assess distributional form. Group-specific boxplots were used to identify systematic residual shifts across distance or viewing-angle groups. Normality was additionally summarized using the Shapiro–Wilk test, and residual variance patterns were evaluated using the Brown–Forsythe test. Observations with absolute normalized residuals greater than three were identified for sensitivity analysis.

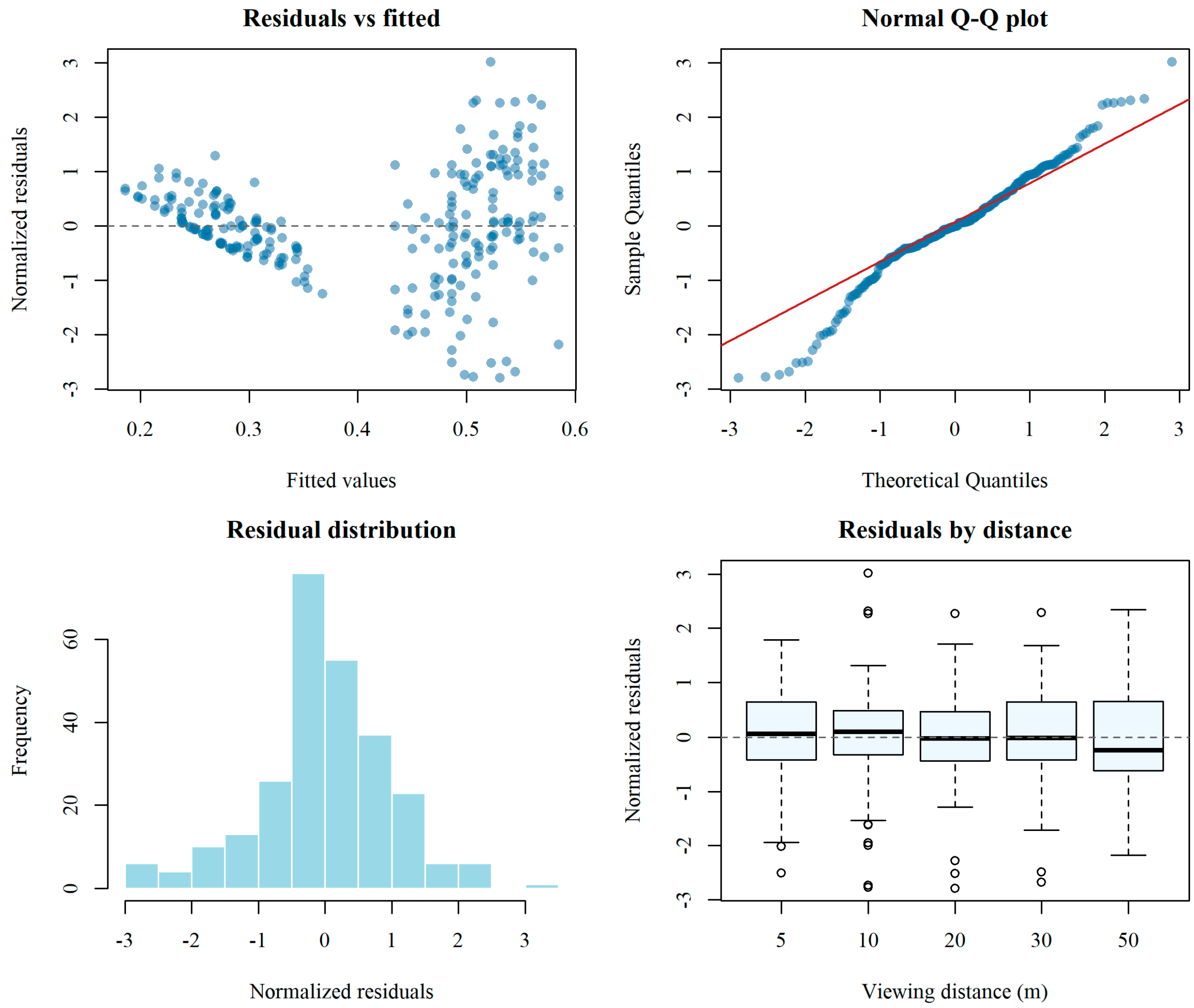

Figure S2. Diagnostic plots for the heteroscedastic linear mixed-effects model used to evaluate the overall distance effect. Panels show normalized residuals against fitted values, a normal Q–Q plot, the residual distribution, and normalized residuals by viewing distance.

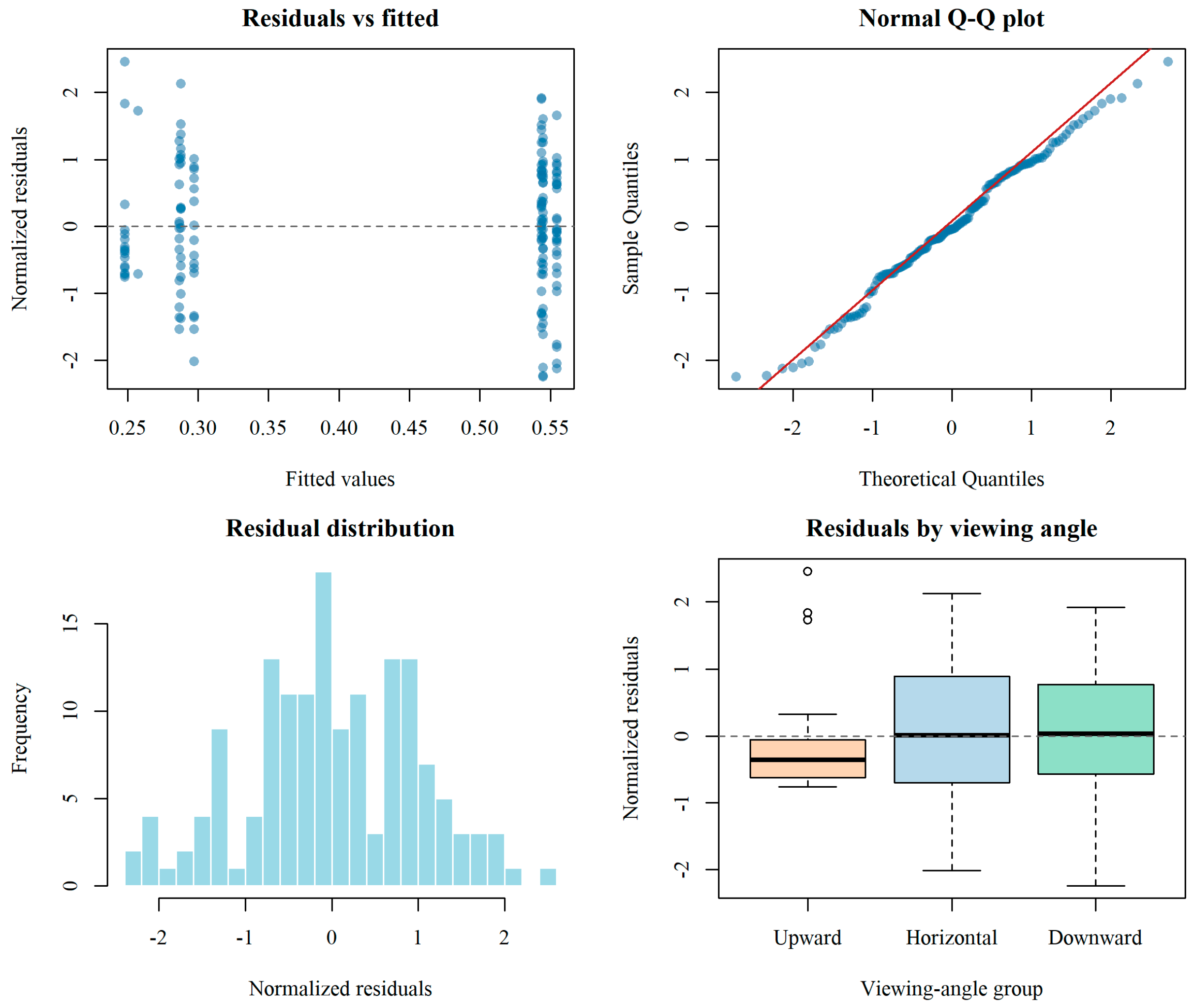

Figure S3. Diagnostic plots for the heteroscedastic linear mixed-effects model used to evaluate the viewing-angle effect at 20–50 m. Panels show normalized residuals against fitted values, a normal Q–Q plot, the residual distribution, and normalized residuals by viewing-angle group.

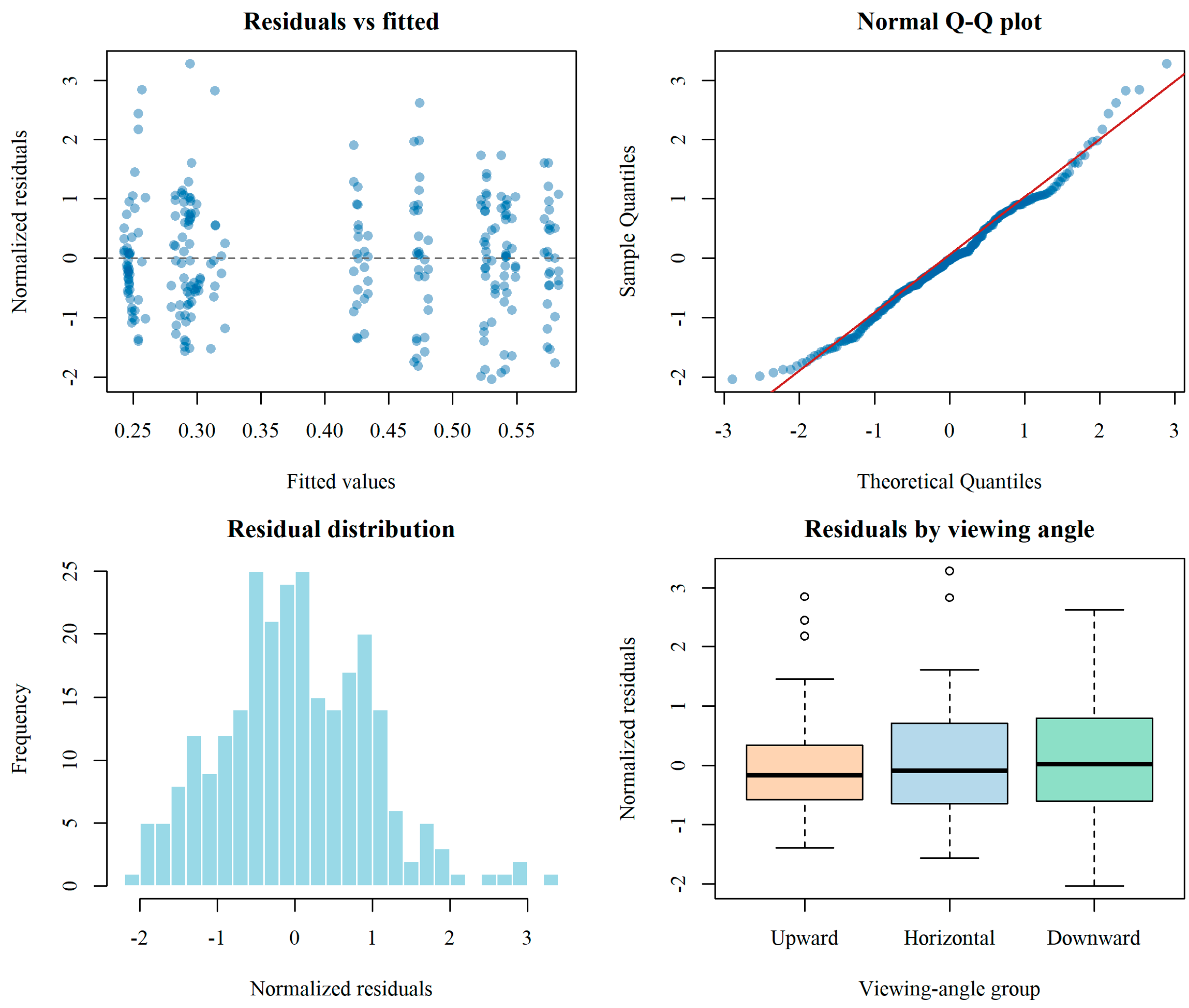

Figure S4. Diagnostic plots for the mixed-effects model used to evaluate the distance × viewing-angle interaction. Panels show normalized residuals against fitted values, a normal Q–Q plot, the residual distribution, and normalized residuals by viewing-angle group.

S4. Representative processed multispectral images

Representative pseudocolored images are provided to illustrate variation in target–background appearance among the processed multispectral scenes (Figure S5).

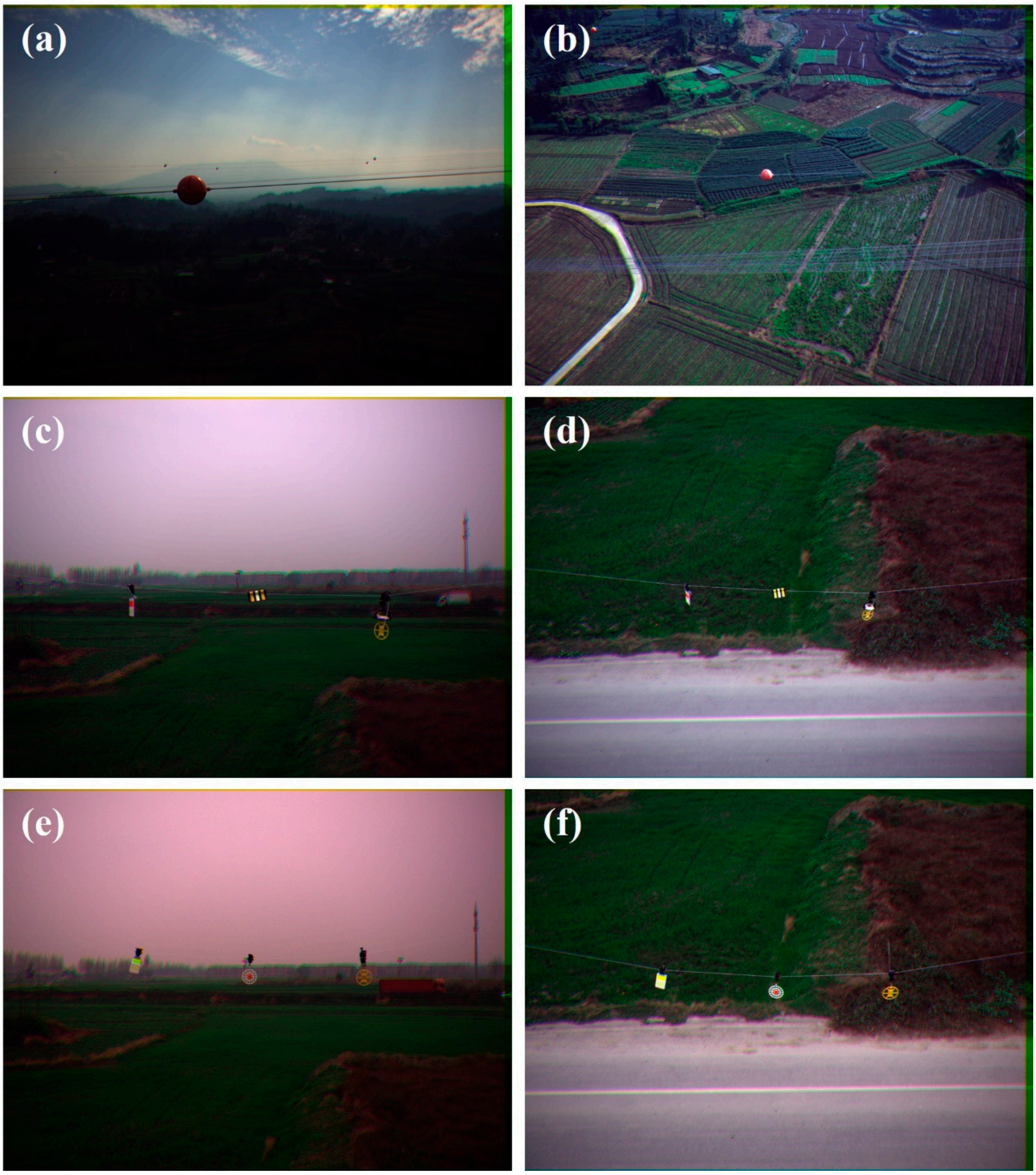

Figure S5. Representative pseudocolored images of warning devices following multispectral processing. The displayed colors visualize differences in the processed spectral signals and do not represent subjective avian color appearance.
